# Sodium Valproate Inhibits the Growth of Human Cholangiocarcinoma *In Vitro* and *In Vivo*

**DOI:** 10.1155/2013/374593

**Published:** 2013-11-13

**Authors:** Bing Wang, Rui Yang, Yue Wu, Hongbo Li, Zouxiao Hu, Yongjun Chen, Shengquan Zou

**Affiliations:** Department of General Surgery, Tongji Hospital, Tongji Medical College of Huazhong University of Science and Technology, Wuhan 430030, China

## Abstract

*Background*. None of treatment options for Cholangiocarcinoma (CCA), including surgery, adjuvant radiotherapy and chemotherapy, and ultimately liver transplantation, have been shown to substantially improve the survival rate in patients with CCA. Valproic acid (VPA), a histone deacetylase inhibitor, has been shown to display potent antitumor effects. In this study, sodium valproate, the clinically available form of VPA, was tested for its ability to inhibit the growth of cholangiocarcinoma cells, both *in vitro* and *in vivo. Materials and Methods.* Cholangiocarcinoma cells (TFK-1, QBC939, and CCLP1) of different origins were treated with sodium valproate to determine their effects on cell proliferation and differentiation, cell cycle regulation, apoptosis, and autophagy. The *in vivo* effects of sodium valproate on cholangiocarcinoma growth were assessed using a xenograft mouse model injected with TFK-1 cells. *Results*. Sodium valproate inhibited cholangiocarcinoma cell growth by inducing cell cycle arrest, cell differentiation, and apoptosis; sodium valproate effects were independent of autophagy. Tumor growth inhibition was also observed *in vivo* using TFK-1 xenografts. *Conclusion*. The *in vitro* and *in vivo* outcomes provide preclinical rationale for clinical evaluation of sodium valproate, alone or in combination with other drugs, to improve patient outcome in cholangiocarcinoma.

## 1. Introduction

Cholangiocarcinoma (CCA) is a highly aggressive malignancy with features of biliary epithelial differentiation, which arises from the epithelia lining the intrahepatic or the extrahepatic bile ducts. Recent data from the USA and UK suggest that the worldwide incidence and mortality from CCA appears to be increasing over the past few decades [1]. The prognosis of CCA is poor because most tumors are advanced at the time of diagnosis. Although several improved therapeutic modalities have emerged and new-targeted therapies are being developed, surgery is the only curative treatment for patients with CCA. Unfortunately, less than one-third of tumors are resectable at diagnosis [2–4]. 5-year survival rates following resection of intrahepatic CCA, distal extrahepatic CCA, and hilar tumors are 22–44%, 27–37%, and 11–41%, respectively [2, 4]. Thus, novel therapeutic approaches need to be developed for the successful treatment of CCA.

**Figure 1:**
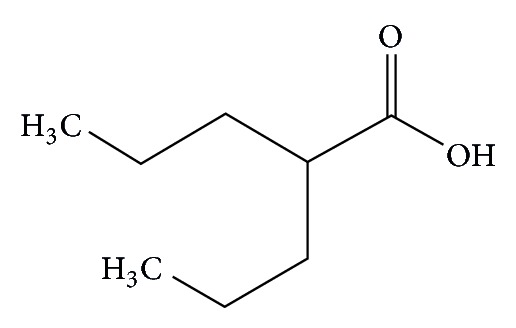
Chemical structure of valproate (VPA, 2-propylpentanoic acid).

Histone deacetylase (HDAC) inhibitors are a class of molecules that modify chromatin structure and regulate gene transcription and expression [5]. Valproic acid (VPA) (Figure 1), a HDAC inhibitor, exerts its primary action by targeting the enzyme HDAC [6, 7]. Sodium valproate is the clinically available form of VPA and is one of the most frequently prescribed antiepileptic drugs [8]. VPA is employed clinically in the treatment of schizophrenia, bipolar disorders, and different forms of headaches. It is also currently under experimental and clinical investigation as an anticancer drug [9]. VPA has shown potent antitumor effects in a variety of *in vitro* and *in vivo* systems, like glioma [10], breast [11], colon [12, 13], prostate [14, 15], and hepatoma [16, 17]. VPA modulates the behavior of various tumor cells by affecting multiple pathways including cell cycle arrest, apoptosis, angiogenesis, metastasis, differentiation, and senescence [18]. VPA is commonly evaluated, either alone or in combination with other agents, in the treatment of hematological malignancies [19]. There is, however, an increasing interest for VPA testing in solid tumors [20, 21], including phase I and phase II clinical studies. Currently, there have been few studies on the growth inhibitory effect of sodium valproate on CCA *in vitro* and *in vivo*. 

The objective of the present study was to demonstrate that sodium valproate inhibits CCA growth both *in vitro* and *in vivo*. Using TFK-1, QBC939, and CCLP1 cells, we evaluated the effects of sodium valproate on cell proliferation, cell differentiation, cell cycle arrest, apoptosis, and autophagy, which enable further insight into the possible mechanisms of sodium valproate. *In vivo* studies used an athymic nude mouse model bearing xenografts of TFK-1 cells, with sodium valproate at a concentration of 300 mg/kg [22], to determine whether sodium valproate inhibits the growth of CCA xenografts. 

## 2. Material and Methods

### 2.1. Cell Culture and Reagents

The human cholangiocarcinoma cell lines, TFK-1 and CCLP1, were purchased from an official cell bank (DSMZ, Germany). QBC939 cells were kindly provided by Dr. Shuguang Wang (the Third Military Medical University, China). TFK-1 cells were originally obtained from a human extrahepatic bile duct [23]. CCLP1 cells were obtained from a peripheral cholangiocarcinoma [24]. QBC939 cells were obtained from intrahepatic cholangiocarcinomas. TFK-1 [25] and QBC939 cells [26] were cultured and maintained in a humidified atmosphere containing 5% CO_2_ at 37°C in RPMI 1640 (GIBCO, Life Technologies, Grand Island, NY), supplemented with 10% fetal bovine serum (GIBCO). CCLP1 [27] cells were cultured and maintained in a humidified atmosphere containing 5% CO_2_ at 37°C in Dulbecco's Modified Eagle Medium (DMEM and GIBCO), supplemented with 10% fetal bovine serum (GIBCO), 2 mM L-glutamine, and 50 *μ*g/mL gentamicin. Sodium valproate was obtained from Sigma-Aldrich (St' louis, MO) and then dissolved in water to a final concentration of 20 mmol/L. Aliquots were stored at −20°C until use to avoid multiple freeze thaw cycles. 

### 2.2. Measurement of Cell Growth and Viability

The growth of cholangiocarcinoma cell lines was determined using a Cell Counting Kit-8 (CCK-8) assay according to the instructions of the manufacturer (Dojindo, Japan). TFK-1 cells (1 × 10^4^), QBC939 cells (5 × 10^3^) and CCLP1 cells (5 × 10^3^) were plated in 6 replicates, cultured for 24 h in 96-well plates, and subsequently treated with sodium valproate at the indicated concentrations up to 120 h. Culture media containing the selected inhibitor was changed daily. After the incubation of the above cell lines with the indicated concentrations of sodium valproate, CCK-8 solution was added at a final concentration of 10 *μ*L/100 *μ*L medium and incubated for an additional 2 h at 37°C. The sample absorbance at 450 nm was determined using a scanning spectrophotometer (Thermo, USA).

### 2.3. Cell Cycle Analysis

Briefly, cells were grown for 24 h and treated with the indicated concentrations of sodium valproate for 24 h, 72 h, or 120 h. Cell cycle analysis was assessed by staining with propidium iodide (PI) and examined by flow cytometry (Becton Dickinson, Franklin Lakes, NJ). 

### 2.4. Detection of Cell Differentiation

TFK-1 and QBC939 cells in the exponential phase were treated with 0, 0.5, and 2 mM sodium valproate. After 120 h of incubation, cell morphology was analyzed microscopically.

### 2.5. Measurement of Apoptosis Induction

TFK-1 cells were treated with sodium valproate (0–8 mM) for 120 h without changing the medium. Apoptosis was determined by a dual-color flow cytometric (FCM) procedure featuring fluorescence isothiocyanate (FITC)-Annexin-V and propidium iodide (PI) staining, according to an Apoptosis Detection Kit (KeyGen, China). 

### 2.6. Hoechst 33342/PI Staining

TFK-1 cells were exposed to sodium valproate (2 mM) for 120 h. The morphology of the apoptotic cells was observed under a fluorescence microscope after being stained with Hoechst 33342/PI (KeyGen). 

### 2.7. Detection of Autophagy with Green Fluorescence Protein-Tagged MAP-LC3

TFK-1 and QBC939 cells were treated with sodium valproate (2 mM) for 3 days. On day 3, the cells were transfected with a green fluorescence protein (GFP)-tagged MAP-LC3 (GFP-LC3) plasmid. After 24 h, the cells were fixed in 4% paraformaldehyde for 30 minutes and mounted for confocal microscopy (Carl Zeiss, Germany). GFP-LC3 expression was used as the characteristic parameter for autophagy. GFP fluorescence was observed via confocal microscopy.

### 2.8. Growth of Cholangiocarcinoma Xenografts in Nude Mice

Evaluation of sodium valproate-induced effects *in vivo* was conducted using xenografts of TFK-1 cells in 6-week-old male Balb-c nu/nu mice with a median weight of 14~16 g. All animal experiments were carried out according to protocols approved by the Experimental Animal Center of Huazhong University of Science and Technology. Ten mice were divided into two treatment groups. All mice had 2 × 10^6^ TFK-1 cells transplanted subcutaneously into the upper-right flank. Treatment was started two weeks after implantation, at which point the tumors were palpable. The mice were injected intraperitoneally with (1) vehicle (control group) or (2) sodium valproate (300 mg/kg BW) every day. Treatment was continued for 14 days. Tumor size was measured 3 times per week and tumor volume was calculated according to the formula: volume (*V*) = *π*/6 × length × width^2^. The mice were evaluated daily for morbidity and mortality. 

### 2.9. Statistical Analysis

All *in vitro* and *in vivo* experiments were repeated in triplicate. Wilcoxon-Mann-Whitney-Test was performed to determine the level of significance for the *in vitro* studies. For *in vivo* studies, the statistical significance was analyzed using the long-rank test. All results were expressed as the mean ± SD. Significance was assumed at *P* < 0.05.

## 3. Results

### 3.1. Effects of Sodium Valproate on Growth of CCA Cells

**Figure 2:**
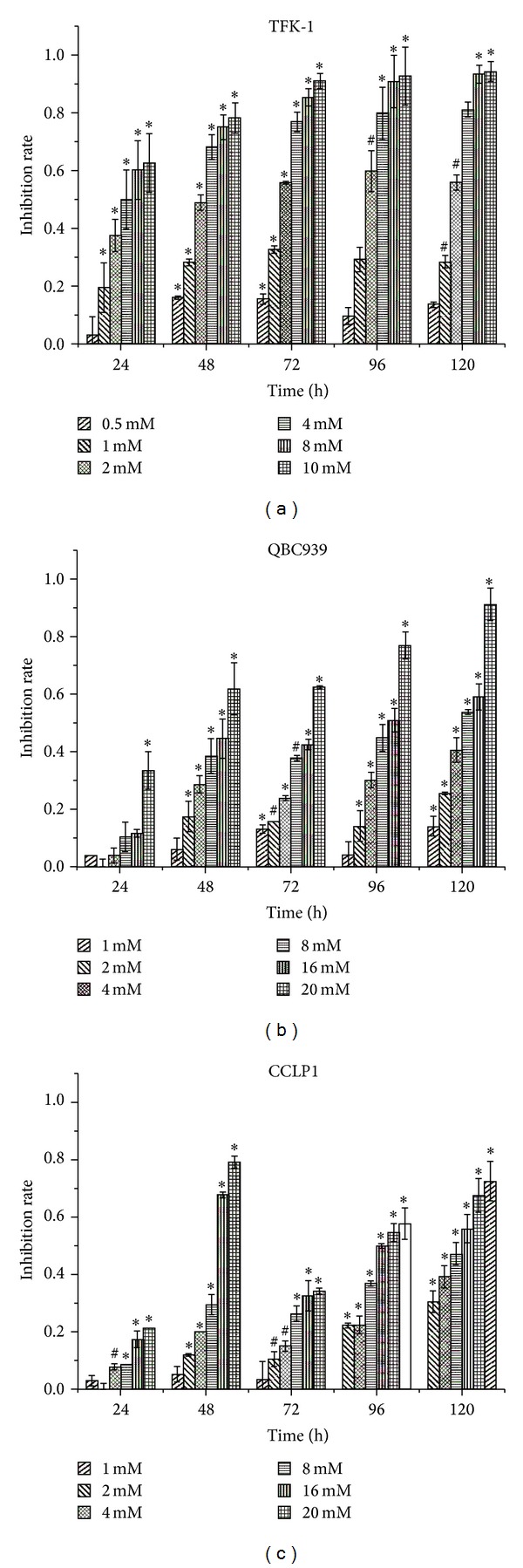
Effects of sodium valproate on cell growth of cholangiocarcinoma cell lines. After exposure to sodium valproate for 24 h–120 h, cell inhibition of TFK-1 (a), QBC939 (b), and CCLP1 (c) was measured by CCK-8 assay. All assays were conducted at least in triplicate. The inhibition rate was in comparison with untreated cells. *P < 0.05 and ^#^ *P* < 0.01.

The CCA cell lines TFK-1 (0–10 mM), QBC939 (0–20 mM), and CCLP1 (0–20 mM) were cultured up to 120 h with various concentrations of sodium valproate, and cell proliferation was assessed by CCK8. Sodium valproate inhibited the proliferation of all the three cell lines in a time- and dose-dependent manner (*P* < 0.05) (Figure 2). We have demonstrated that TFK-1 cells were more sensitive to sodium valproate than the other two cell lines. QBC939 cells showed almost identical proliferation characteristics compared to CCLP1 cells. In TFK-1 cells, treatment with 2 mM sodium valproate for 72 h resulted in >50% suppression of cell proliferation (Figure 2(a)), whereas in QBC939 cells and CCLP1 cells, a 50% suppression required exposure to 8 mM sodium valproate for 120 h (Figures 2(b) and 2(c)). Further experiments were restricted to TFK-1 and/or QBC939 as the representative cell lines. 

### 3.2. Inductive Effect of Sodium Valproate on TFK-1 Cell Line Differentiation

**Figure 3:**
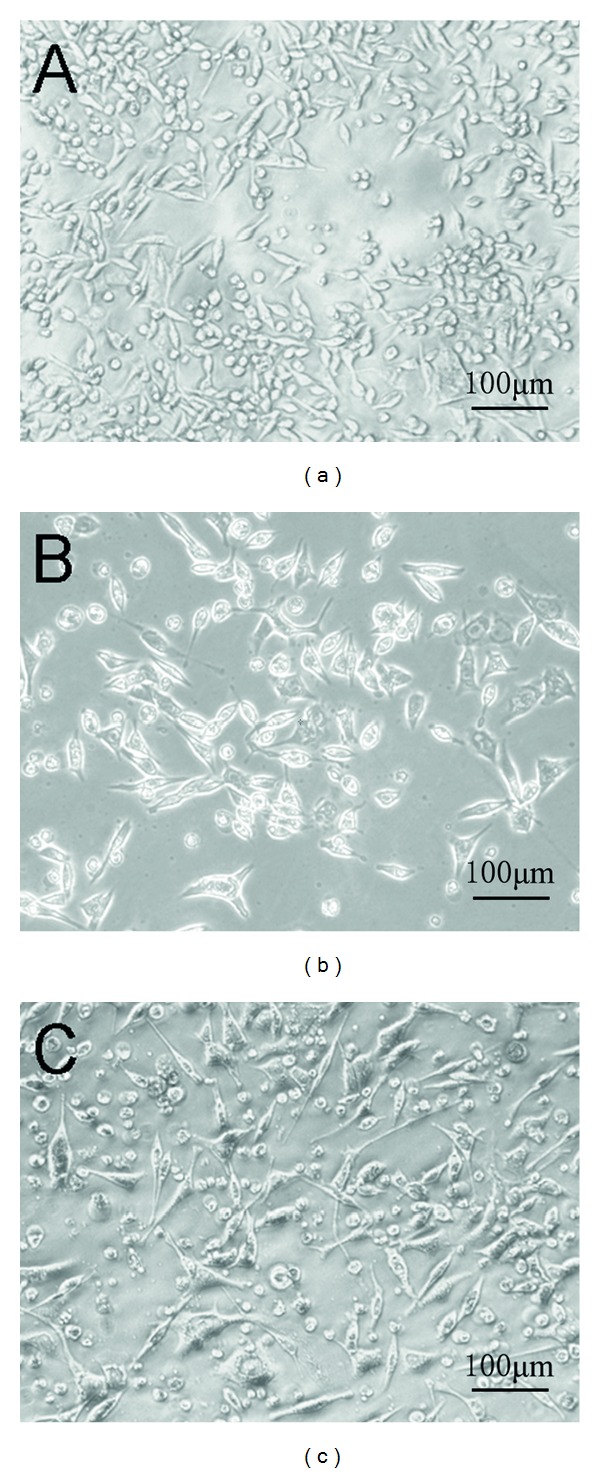
Inductive effect of sodium valproate on TFK-1 cell differentiation. Dendrite-like structure formation in TFK-1 cells treated for 120 h with (a) 0 mM (control), (b) 0.5 mM, and (c) 2 mM sodium valproate. With the increase of sodium valproate concentration, the dendrite-like structures became progressively longer. The morphology of cells was analyzed using a Nikon FX-35A camera (200x magnification). Each experiment was performed in triplicate.

The differentiation status of CCA cells was monitored by the formation of dendrite-like cellular protrusions. As shown in Figure 3, with the treatment of 0–2 mM sodium valproate for 120 h, TFK-1 cells differentiated into dendrite-like structures. These dendrite-like structures became progressively longer in accordance with the increase in sodium valproate concentration. The data suggests that sodium valproate induces TFK-1 cells to differentiate *in vitro*. Sodium valproate at the same concentration showed no changes in differentiation of QBC939 cells (data not shown). 

### 3.3. Induction of Apoptosis in TFK-1 Cells by Sodium Valproate

**Figure 4:**
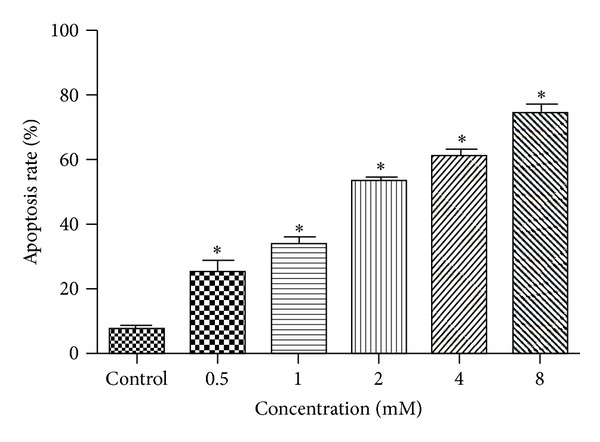
Induction of apoptosis in TFK-1 cells by sodium valproate. TFK-1 cells were incubated with 0–8 mM sodium valproate for 120 h. Apoptosis was measured by Annexin-V and PI double staining. The values represent the mean ± S.D. (*n* = 3). *P < 0.05 versus control.

To examine whether the antiproliferative effects of sodium valproate are due to the induction of apoptosis, TFK-1 cells were incubated with 0–8 mM for 120 h, then stained with Annexin-V and PI, and analyzed via flow cytometry. Sodium valproate showed a strong dose-dependent induction of apoptosis in TFK-1 cells (Figure 4). 0.5 mM sodium valproate induced an apoptosis rate of 23% and 8 mM induced apoptosis in 70% of TFK-1 cells (Figure 4).

**Figure 5:**
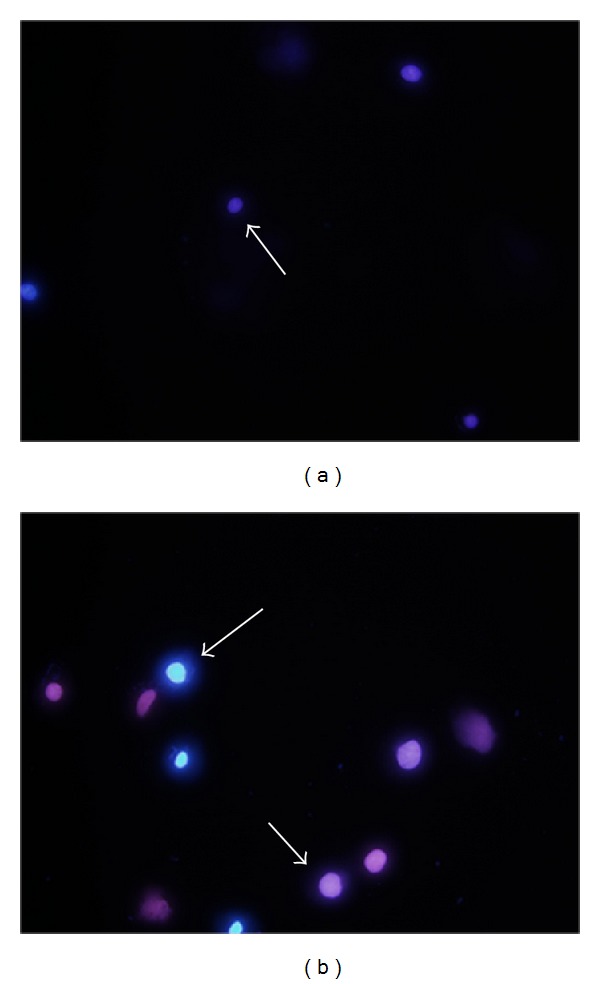
Apoptotic morphological changes in TFK-1 cells after treatment with sodium valproate. After 120 h, untreated control group (a) and 2 mM sodium valproate-treated TFK-1 cells (b) were loaded with Hoechst 33342/PI and then observed via fluorescence microscopy (100x magnification). Mixture of low blue with low pink fluorescence indicates live cells ((a), short arrow), while high blue fluorescence indicates apoptotic cells ((b), long arrow), and pink represents dead cells ((b), short arrow).

The apoptotic morphological changes of TFK-1 cells were observed via fluorescence microscopy using Hoechst 33342/PI staining after exposure to 2 mM sodium valproate for 120 h. In contrast to the untreated group, there were higher percentages of bright blue cells (apoptosis) in the sodium valproate-treated group (Figure 5). 

### 3.4. Cell Cycle Arrest by Sodium Valproate in Cell Cycle Distribution

**Figure 6:**
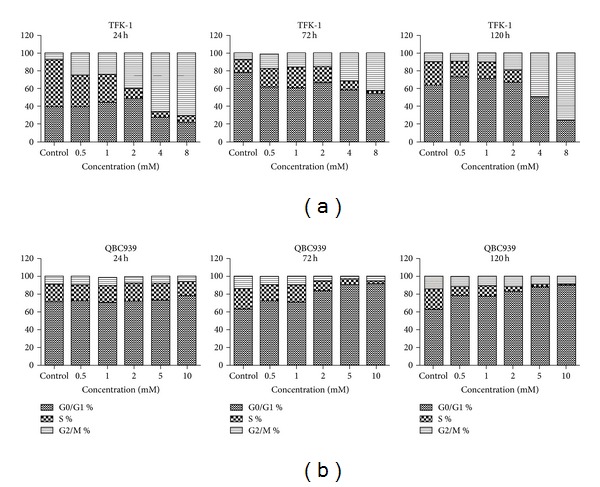
Effects of sodium valproate on cell cycle distribution in cholangiocarcinoma TFK-1 cells and QBC939 cells. (a) TFK-1 cells were incubated with 0–8 mM sodium valproate, and (b) QBC939 cells were incubated with 0–10 mM sodium valproate. On 24 h, 72 h, and 120 h, cells were harvested, and the fractions of cells in G0/G1-phase, S-phase, and G2/M-phase were determined by flow cytometry.

To determine whether the antiproliferative effects of sodium valproate on CCA growth results from inhibition of cell cycle, cell cycle analyses were performed on TFK-1 cells and QBC939 cells after exposure to sodium valproate for 24, 72, and 120 h. As shown in Figure 6, TFK-1 cells were arrested in the G2/M phase in a dose-dependent manner after sodium valproate treatment for 24 h, 72 h, and 120 h (Figure 6(a)). With increasing concentration of sodium valproate, the percentage of cells in the G2/M phase increased from 7.78% to 71.17% at 24 h, 12% to 42.53% at 72 h, and 10.72% to 75.57% at 120 h. This concentration-dependent accumulation of cells in the G2/M phase was accompanied by a decrease in the number of cells in G0/G1 phase and S phase (Figure 6(a)). In contrast, QBC939 cells displayed an increase in the G0/G1 fraction of cells with the increase of sodium valproate concentration (Figure 6(b)). With an increasing concentration of sodium valproate, the percentage of G0/G1 cells increased from 71.42% to 74.04% at 24 h, 63.07% to 91.82% at 72 h, and 63.07% to 90.27% at 120 h. At the same time, with the accumulation of QBC939 cells in the G0/G1 phase, there was a concomitant decrease in cells within S phase and a slight decrease in cells within the G2/M phase (Figure 6(b)).

### 3.5. Effect of Sodium Valproate on Autophagy

**Figure 7:**
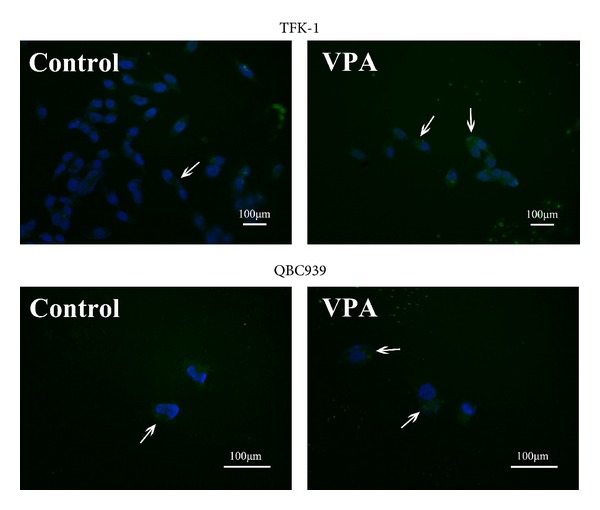
Sodium Valproate induces autophagy in TFK-1 cells and QBC939 cells. TFK-1 cells and QBC939 cells were transfected with green fluorescence protein (GFP) and tagged with MAP-LC3 (GFP-LC3) plasmid and treated with sodium valproate at 2 mM and 8 mM, respectively. The formation of punctate GFP-LC3 spots is indicative of autophagy (short arrow).

To assess whether there is a third possible mechanism that could contribute to the inhibitory effects of sodium valproate, we evaluated the role of autophagic cell death. TFK-1 cells and QBC939 cells were transiently transfected with a GFP-LC3 plasmid, treated with sodium valproate for 3 days, and then assessed for cell death via a confocal fluorescence microscope. The number of GFP-LC3 puncta counted in TFK-1 cells increased from 13 puncta per 100 cells (untreated control) to 21 puncta (2 mM treated TFK-1 cells) (Figures 7(a) and 7(b)), whereas in QBC939 cells, the number of puncta increased from 15 puncta per 100 cells (untreated control) to 26 puncta (8 mM treated QBC939 cells) (Figures 7(c) and 7(d)). Considering these results, although VPA induced autophagic cell death to a certain degree, the autophagy cell rates remained at a low level. 

### 3.6. Sodium Valproate Exhibits Inhibition of CCA Xenograft Growth

**Figure 8:**
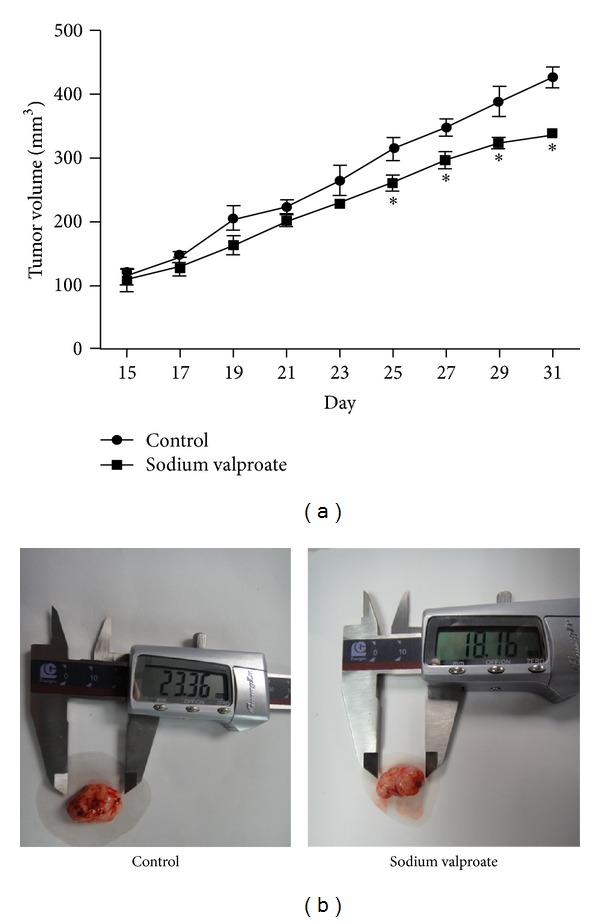
The effect of sodium valproate treatment on cholangiocarcinoma xenografts. (a) Tumor xenografts were established in mice by implanting TFK-1 cells on the upper-right flank. Animals were randomly divided into 2 groups and injected through i.p. with vehicle (control group) or sodium valproate (300 mg/kg, every day) for 2 weeks. Values were presented as mean ± SD. *P < 0.05 compared with the control. (b) Representative tumor tissue excised from control (left) and treated groups (right).

**Figure 9:**
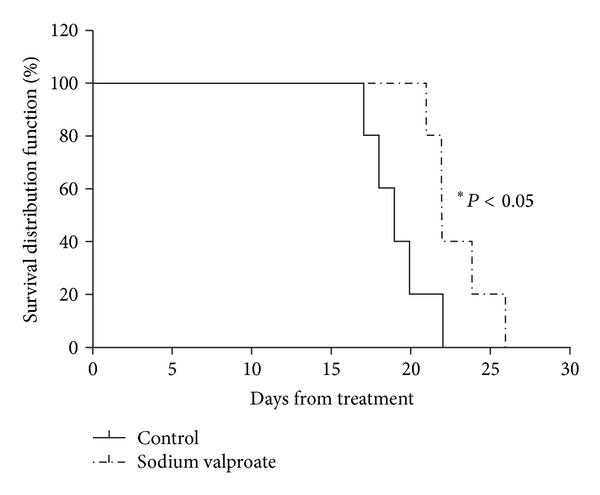
Percentage survival of animals in control and sodium valproate-treated groups. Animals in both sodium valproate-treated and control groups were monitored every day from the first day of treatment until death. By using the Kaplan-Meier method, survival times were determined. The mean percent survival of sodium valproate treated animals was significantly higher, 60% compared to the untreated control, which was 20% over 22 days.

To further validate our *in vitro* studies, we utilized nude mice with subcutaneous human CCA xenografts. We evaluated the effect of sodium valproate treatment on tumor size and survival. As shown in Figure 8, animals treated with sodium valproate (300 mg/kg, injected intraperitoneally daily) showed statistically significant reduction in the tumor volume compared to the control group.  Figure 9 shows the Kaplan-Meier survival curves for each experimental group. Compared to control group, a statistically significant improvement in overall survival was observed in the sodium valproate group. 

## 4. Discussion

 Our previous work had reported that trichostatin A (TSA), an HDAC inhibitor, was effective at suppressing CCA cell growth [26]. Sodium valproate has been shown to have anticancer activity against a variety of tumor types. However, few studies have evaluated the antitumor activity of sodium valproate in CCA growth. In the present study, we found that sodium valproate inhibited the growth of CCA. CCK8 assays showed that CCA cells TFK-1, QBC939, and CCLP1 treated with sodium valproate led to reduced viability (Figure 1), especially with the extension of incubation time and increase in concentration.

Consistent with other solid tumors [28, 29], our data showed sodium valproate induces apoptosis in CCA cell lines. It was observed that TFK-1 cells appeared shrunken, with condensation of both nuclear chromatin and cytoplasm. The results of morphological changes were consistent with results from Annexin-V/PI staining. The finding sodium valproate induces apoptosis of CCA lines is also supported by studies in other gastrointestinal cancer lines. For example, VPA synergizes with TRAIL to induce apoptosis of the pancreatic cancer lines, MiaPaCa2, and Panc1 [30]. Also, the HDAC inhibitor, valproic acid, induces p53-dependent radiosensitization in colon cancer cells [31]. 

 Over the past ten years, there have been many studies on whether or not drugs inducing differentiation* in vitro* are effective in the treatment of patients with specific types of cancer. Differentiation was typically monitored by either morphological, enzymatic, or biochemical means [32]. Dendrite-like structure is a quantifiable marker of CCA cell differentiation. Our present studies demonstrated that sodium valproate induces TFK-1 cells to undergo terminal differentiation. More dendrite-like structures were observed with the extension of incubation time and the increase in sodium valproate concentration. Once the cells begin terminal differentiation, it is common that cell division and cell proliferation ceases. In a sense, sodium valproate can suppress the malignant phenotype of TFK-1 cells. Therefore, terminal differentiation of TFK-1 cells induced by sodium valproate should be important in inhibiting CCA cell proliferation *in vitro. *In line with our data, valproate affects differentiation and decreases proliferation of endometrial stromal sarcoma cells [33]. Also, in uveal melanoma cells, VPA induces cell growth arrest and differentiation [34].

One of the most common ways for inhibiting proliferation of tumor cells using antineoplastic agents is through their role in cell cycle arrest. Our present research provided experimental evidence that the antitumor effect of sodium valproate on TFK-1 and QBC939 cells is associated predominantly with cell cycle arrest. Other studies have reported that cells arrest at the G2/M phase after exposure to VPA [35, 36]. In line with this research, our experiments showed that the numbers of TFK-1 cells at the G2/M phase increased with the increase of sodium valproate concentration. Interestingly, QBC939 cells were arrested at the G0/G1 phase, but not at the G2/M phase. The reasons for this difference remain unclear and may warrant further mechanistic studies. However, in both cell lines, the dose-dependent decrease in S phase cells was observed with the increase of sodium valproate concentration.

To our knowledge, the induction of autophagy in CCA cells by sodium valproate has not been studied previously. In our study, although sodium valproate causes autophagy in CCA cells to a certain degree, the total autophagy rates remained at a relatively low level. Therefore, it can be concluded that autophagy may not be a primary mechanism by which sodium valproate induces cytotoxic effects in CCA cells.

A total of 300 mg/kg daily of sodium valproate over 2 weeks significantly reduced the growth of xenografted TFK-1 cells by 20.73%, which confirmed our *in vitro* data. However, a different VPA regimen may be required to treat other tumor types. Daily i.p. injections of 366 mg/kg VPA were necessary to inhibit gastrointestinal tumor growth in nu/nu mice [37], and neuroblastoma xenograft studies were based on 400 mg/kg VPA [38]. We also planned our animal experiments to evaluate the effect of sodium valproate treatment on survival of the animals and used the Kaplan-Meier method to calculate the mean percent survival time in both treatment and control groups. In our study, we discovered that the mice treated with sodium valproate suffer from comparatively lesser tumor burden and survive longer than the ones of the control groups.

In summary, the results from this study demonstrate that sodium valproate is capable of suppressing CCA cell growth both *in vitro* and *in vivo* and may provide a therapeutic benefit for treating CCA. However, there is still much to be studied regarding the molecular mechanism by which VPA induces differentiation, cell cycle arrest, and apoptosis. In addition, further studies of VPA in combination with classical chemotherapeutic drugs are necessary for a better understanding of CCA development/progression, which might lead to further clinical application of VPA in patients with liver diseases.
